# The Provings of Natrum Muriaticum

**Published:** 1890-09

**Authors:** A. McNeil

**Affiliations:** San Francisco, Cal.


### THE PROVINGS OF NATRUM MURIATICUM.

A. McNeil, M. D., San Francisco, Cal.

On page 257, vol. X, of The Homœopathic Physician are the following words, written by me: " Dr. Watzke, of Vienna, undertook to prove this drug (Natrum muriaticum), and, in spite of his pre-conceived opinions, was compelled to acknowledge that not till he went up to the high potencies could he produce symptoms in the healthy of any value." (I quote from memory).

I afterward received a letter from Dr. Richard Hughes, of Brighton, England, referring me to an article of his in the *Hahnemannian Monthly* for December, 1889, and very courteously intimating that my statement at the beginning of this article conveyed a false impression which his paper made clear. I am always willing to make amends when I have done any one an injustice. And in order that all may see whether or not I erred, I place Dr. Watzke's statement on the question, as translated by Carrol Dunham in vol. II, "Transactions of the New York Homœopathic Society," and which is also quoted by Dr. Hughes in the above-mentioned article, in juxtaposition with the Doctor's statement.

| Dr. Watzke says: | Dr. Hughes says: |
| --- | --- |
| "I am alas! I say alas! for I would much rather have upheld the larger doses, which accord with current views: I am compelled to declare myself for the higher dilutions. The physiological experiments made with Natrum muriaticum, as well as the great majority of the clinical results obtained therewith speak decidedly and distinctly for those preparations." | "The general account to be given of them (the Austrian reprovings of Nat-mur.), is that under the higher potencies there was little genuine disturbance of health; that the activity of the drug as the provers went lower showed itself greater when the first triturations and small doses of the crude substance were being taken. There are exceptions of course to this statement, but I think you will find that they are exceptions, and that what I have said is the rule." |
| Dr. Hughes says, *loc. cit.*, page 772, of the Austrian reproving of Natrum muriaticum: "No pains were spared to test the drug in every form from the 30th dilution down to the crude salt." | Dr. Hughes says, page 773: "It may be a question what he (Watzke) means by the 'higher' dilutions. Nowadays we think of the 30th and upwards when we thus speak, but it was not so in 1848." |

